# Supplementary material for: Causal Relationship between Aging and Anorexia Nervosa: A White-Matter-Microstructure-Mediated Mendelian Randomization Analysis
Source: Biomedicines. 2024 Aug 16;12(8):1874. doi: 10.3390/biomedicines12081874 (PMC11351342; doi:10.3390/biomedicines12081874)
Supplement: Supplementary file 1 [file biomedicines-12-01874-s001.zip › Supplementary Figure/Figure S4/Singe SNP plot of MR analysis for TL to ubm-b-1817.pdf]

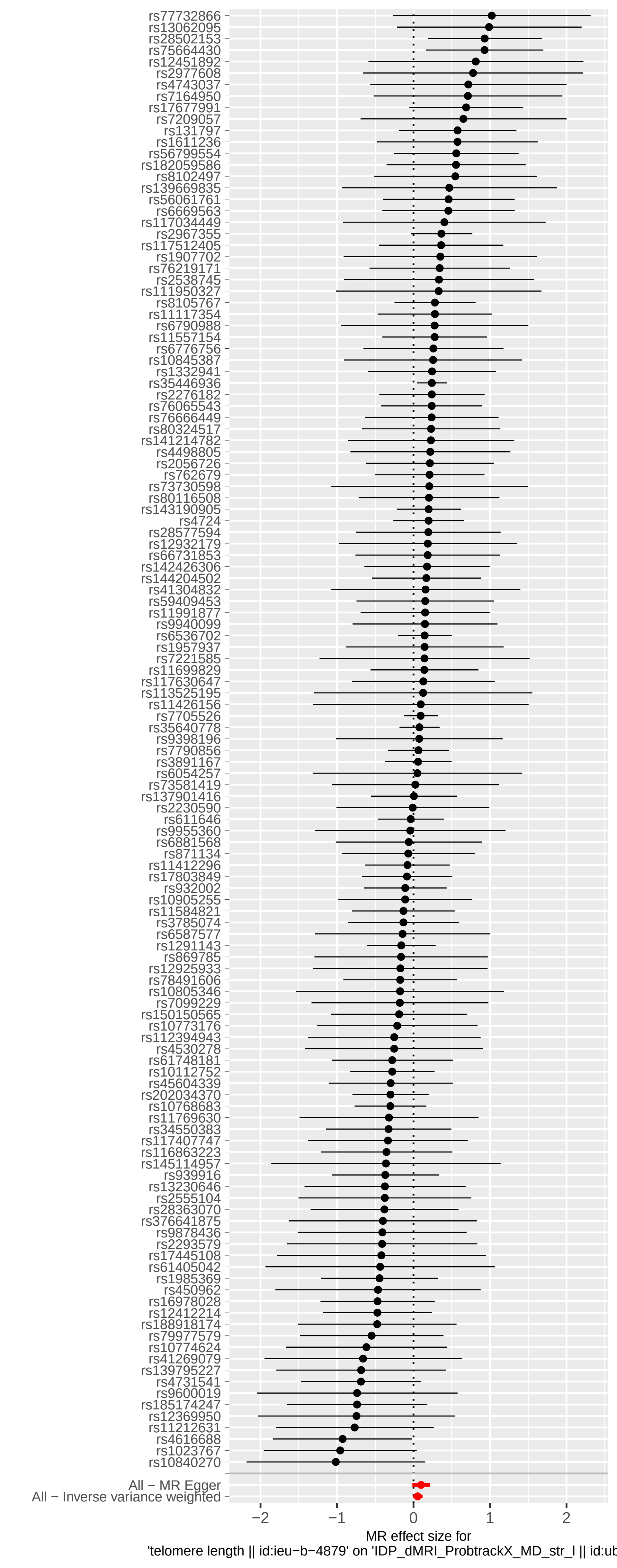

rs77732866
rs13062095
rs28502153
rs75664430
rs12451892
rs2977608
rs4743037
rs7164950
rs17677991
rs7209057
rs131797
rs1611236
rs56799554
rs182059586
rs8102497
rs139669835
rs56061761
rs6669563
rs117034449
rs2967355
rs117512405
rs1907702
rs76219171
rs2538745
rs111950327
rs8105767
rs11117354
rs6790988
rs11557154
rs6776756
rs10845387
rs1332941
rs35446936
rs2276182
rs76065543
rs76666449
rs80324517
rs141214782
rs4498805
rs2056726
rs762679
rs73730598
rs80116508
rs143190905
rs4724
rs28577594
rs12932179
rs66731853
rs142426306
rs144204502
rs41304832
rs59409453
rs11991877
rs9940099
rs6536702
rs1957937
rs7221585
rs11699829
rs117630647
rs113525195
rs11426156
rs7705526
rs35640778
rs9398196
rs7790856
rs3891167
rs6054257
rs73581419
rs137901416
rs2230590
rs611646
rs9955360
rs6881568
rs871134
rs11412296
rs17803849
rs932002
rs10905255
rs11584821
rs3785074
rs6587577
rs1291143
rs869785
rs12925933
rs78491606
rs10805346
rs7099229
rs150150565
rs10773176
rs112394943
rs4530278
rs61748181
rs10112752
rs45604339
rs202034370
rs10768683
rs11769630
rs34550383
rs117407747
rs116863223
rs145114957
rs939916
rs13230646
rs2555104
rs28363070
rs376641875
rs9878436
rs2293579
rs17445108
rs61405042
rs1985369
rs450962
rs16978028
rs12412214
rs188918174
rs79977579
rs10774624
rs41269079
rs139795227
rs4731541
rs9600019
rs185174247
rs12369950
rs11212631
rs4616688
rs1023767
rs10840270
All − MR Egger
All − Inverse variance weighted
−2
−1
0
1
2
MR effect size for
'telomere length || id:ieu−b−4879' on 'IDP_dMRI_ProbtrackX_MD_str_l || id:ub
